# Reviewed article: Couto VDCS, Vanhoz ABL, Gabe KT, Oliveira CZ, Jaime PC. Self-efficacy to provide dietary guidance based on the Brazilian Dietary Guidelines: An Uncontrolled Community Trial. Epidemiol Serv Saude. 2025:34;e20240330

**DOI:** 10.1590/S2237-96222025v34e20240330.a

**Published:** 2025-06-13

**Authors:** Angélica Ribeira e Silva

**Affiliations:** Universidade Federal de Minas Gerais, Nutrição

**Table:** 

Rating	Excellent	Good	Average	Below average	Poor
Interest	✓				
Quality			✓		
Originality		✓			
Overall		✓			

**Table:** 

Questionnaire	Yes	No	Not applicable
Does the manuscript contain new and significant information to justify publication?	✓		
Does the Abstract (Summary) clearly and accurately describe the content of the article?	✓		
Is the problem significant and concisely stated?	✓		
Are the methods described comprehensively?	✓		
Are the interpretations and conclusions justified by the results?	✓		
Is adequate reference made to other work in the field?	✓		

## Manuscript Structure

Length of article is: Adequate

Number of tables is: Adequate

Number of figures is: Adequate

Please state any conflict(s) of interest that you have in relation to the review of this paper (state “none” if this is not applicable): None

## Comments

Parabenizo pelo trabalho, ele é de exterma importância para o fortalecimento da APS. Mais relevante ainda é saber que o curso está disponível para a população brasileira. Coloquei contribuições no documento em anexo.

Atenciosamente. 

